# Animal and environmental risk factors for sporadic Shiga toxin-producing Escherichia coli (STEC) infection in England: a case control study for O157, O26 and other STEC serotypes

**DOI:** 10.1080/20477724.2023.2197672

**Published:** 2023-04-04

**Authors:** Erica Kintz, Julii Brainard, Mike Vanderes, Roberto Vivancos, Lisa Byrne, Saira Butt, Claire Jenkins, Richard Elson, Iain Lake, Paul Hunter

**Affiliations:** a Norwich Medical School, University of East Anglia, Norwich, UK; b NIHR Health Protection Research Unit in Gastrointestinal Infections, University of Liverpool, Liverpool, UK; c NIHR Health Protection Research Unit in Emergency Preparedness, University of East Anglia, Norwich, UK; d Field Epidemiology Services, UK Health Security Agency, Liverpool, UK; e NIHR Health Protection Research Unit in Emerging and Zoonotic Infections, University of Liverpool, Liverpool, UK; f Gastrointestinal Pathogens Unit, UK Health Security Agency, London, UK; g School of Environmental Sciences, University of East Anglia, Norwich, UK

**Keywords:** Shiga toxin E. coli, logistic models, risk factors, case-control studies

## Abstract

Most Shiga toxin-producing *E. coli* (STEC) infections are sporadic. Routine enhanced surveillance questionnaires of confirmed STEC cases in England contained promising data to conduct a case-control study to identify non-food exposures linked to the risk of becoming infected with different STEC serotypes, including O157, O26 and all others; this study pulled eligible cases from the recorded enhanced surveillance data. Controls were recruited from the general population and answered a comparable postal questionnaire. Logistic regression was performed to identify risk factors associated with STEC infection for O157, O26 and other serotype cases. In adjusted models, travel outside of the U.K. and childcare occupations raised the risk of infection for all serotypes. Day trips within the UK, exposure to dogs and contact with soil were linked to lower infection risk. Resident region within England was often linked to decreased risk. Summer season was linked to O157 and O26, but not other STEC. Swimming in the sea was linked to increased risk of infection by O157, but not other types of STEC. Correlations between exposures and infection were similar when the analysis was repeated excluding participants with a history of foreign travel. As the first case-control study in England to include sporadic non-O157 STEC, the varying risk factors between O157 and non-O157 cases suggest there are potentially unique reservoirs for different serotypes.

## Introduction

Shiga toxin-producing *E. coli* (STEC) belong to a heterogenous group of gastrointestinal pathogens defined by the presence of one or more bacteriophage-encoded Shiga toxin (*stx*) genes. Shiga-toxin (Stx) binds to the host intestinal epithelium leading to cell death. If this toxin enters the bloodstream, it can affect other organs, like the kidney or brain [1]. STEC cause a wide range of symptoms, including hemorrhagic colitis, involving severe abdominal cramping and bloody diarrhea. Up to 30% of STEC cases require hospitalization, and about 10% of cases develop hemolytic uremic syndrome (HUS). STEC-HUS is a life-threatening systemic condition characterized by renal failure, and sometimes associated with neurological and cardiac complications [2,3].

STEC colonize the gastrointestinal tract of ruminant animals and are part of their normal intestinal flora. People become infected by ingesting small amounts of feces, whether from contaminated foods or coming into direct contact with the animals or their environment. STEC requires a much lower infectious dose compared with many other gastrointestinal bacteria, with an estimated 10–100 organisms capable of causing infection [4,5]. This increases the risk of infection from any single exposure and facilitates person-to-person transmission.

STEC serotype O157:H7 emerged as a threat to public health in the early 1980s after two outbreaks of food poisoning in the U.S.A associated with a chain of fast food restaurants [6]. Shortly thereafter, an increase in the number of cases of HUS in 1982–1983 demonstrated that the pathogen was also present in the U.K. [7]. In England, the number of cases of STEC O157:H7 remained stable from the mid-1990s, at around 800 cases each year through 2015 [2,8]. Since 2015, the number of STEC O157:H7 diagnoses per year have been decreasing, whiereas non-O157 STEC diagnoses have been increasing [9]. The increase in notifications of non-O157 STEC is largely due to more public health laboratories testing for all STEC serotypes using a PCR targeting *stx*, although a genuine rise in incidence cannot be ruled out [3].

Since the 1980s, foodborne outbreaks of STEC O157:H7 in the U.K. were often attributed to contaminated meat and dairy products [10]. Many recent outbreaks were linked to ready-to-eat products, especially salad vegetables [11,12]. In England, between 1983 and 2012, Adams et al. [10] found that STEC O157 outbreaks associated with meat and milk decreased, and outbreaks attributed to petting farms increased. Butt et al. also concluded that environmental factors and/or animal contact are an important risk factor for transmission of STEC, and that environmental contamination from farm animals was a significant driver in the burden of sporadic STEC infection [2].

Most STEC infections are sporadic and are not part of a recognized outbreak [10]. Identifying possible sources of these infections requires performing a case-control study; the last such study carried out in England was in 1997 [13]. Given apparent changes in common sources of infections for outbreaks [10], it is possible that transmission routes for sporadic infections are changing. To identify the most common sources of sporadic STEC infections in England, we undertook an updated case-control study, taking advantage of the National Enhanced Surveillance System for STEC (NESSS) infections maintained by the UK Health Security Agency (UKHSA) since 2009.

## Methods

### Study design

A prospective case-control study was run between February 2019 and March 2020, using UKHSA’s collection of Enhanced Surveillance Questionnaires (ESQs) for notified STEC infections [8]. Participants had to be resident in England and (at time of invitation to participate) age 0–70 years of age. The study received approval for our research methods including recruitment strategy, participant information and consent procedures, secure data management and dissemination plans from the NHS Health Research Authority (REC reference 17/SC/0527) and their Confidentiality Advisory Group (CAG reference 17/CAG/0164).

### Case selection

Case and control data were collected from February 2019 to March 2020. Case data for persons age ≤ 70 at onset of infection were extracted from the National Enhanced Surveillance for STEC database maintained UKHSA [8]. Cases completed the ESQ via a telephone interview with an environmental health practitioner or health protection officer [14]. Cases were excluded if they were part of a recognized outbreak, co-infected with another diarrheal pathogen, resident outside England or in prison. Cases or controls living in other types of group residential facilities, such as care homes or boarding schools, were eligible for inclusion.

### Control selection (recruitment)

Target recruitment was two controls to each case of STEC O157, as many more O157 cases were recorded per year compared to non-O157 cases at the time of protocol development. For control recruitment, NHS Digital provided a database of addresses of randomly selected (using the random function in SQL) individuals resident in England and registered with a NHS primary care provider. NHS primary care providers are part of a national universal health care system that has existed in England since 1948 [15]. In the NHS model, primary care providers manage patient records and access to non-urgent health care (generalist or specialist). Recruitment of controls through NHS primary care patient records had the advantage that registration with a primary care practice is near universal for all age groups [16], while patient demographic information was usually complete (age, sex and usual address). We know of no similarly complete and universal population list for all age groups among English residents.

The list of candidate controls for our study was frequency age matched (in groups shown in Table S1 in Supplementary) to reflect the distribution of ages of STEC O157 cases in 2015 – these data were the most complete available when this study was designed [17]. The majority of historical cases and persons with the most severe illness were fairly young while we expected higher response rates from older adults, so we limited the upper recruitment age for both cases and controls to about 70 years to reduce over-representation of older adults, especially among controls, for a disease that tends to be most harmful to the very young.

Control invitations were only frequency matched by age groups, not other traits. Invitations for participation in the case-control study and the questionnaire were mailed to individuals along with one reminder letter 2 weeks after initial invitation. The control questionnaire was a modified version of the ESQ, collecting the same exposure data. Participants completed the paper questionnaire on their own, considering exposures in the week prior to completing the form, and returned forms to the research team via a postage-paid envelope. Controls were asked for consent and excluded if they had had a gastrointestinal illness, described as upset stomach or diarrhea, in the prior month to completing the questionnaire; 274 contacts replied to the study invitation that they were ineligible for inclusion for this reason. For children aged 0–7, the parent completed the questionnaire and consent form on behalf of the child. For children aged 8–15, an assent form was also completed by the child in addition to the consent form completed by the parent; older children were encouraged to assist in the completion of the questionnaire by the parent.

### Data handling

At the end of the study, UKHSA provided data for eligible cases, with unique identifier, serotype, age and post code. Control responses were entered in a Microsoft Access database using forms to help prevent data entry errors. Date entry was validated by double-checking entry on 10% of the records. Data were cleaned and processed in Microsoft Excel, with models constructed in Stata. Cases and controls were fully anonymized before analysis. Unanswered questions were a recurring feature of the datasets, for both cases and controls. When true/false questions were not completed, these were recorded as false during data cleaning. This is because many control respondents only recorded positive responses and left other fields blank as opposed to recording ‘no’; this strategy has been used previously to deal with missing data [18,19]. No cases or controls were excluded from the study at this stage due to incomplete questionnaires. Missing data were not imputed for other (non-binary answer) fields because the omissions were typically not missing at random but rather absent depending on case/control status. For information on how exposures were defined, please see the controls questionnaire in Supplementary.

All exposures and potential confounders were expressed categorically, such as ‘Season that questionnaire was completed’ (4 levels, March–May = spring, June–August = summer, September–November = fall, December–February = winter. Participant home postcodes were linked to the lower super output area (LSOA) where they resided, using look up tables available from the Office for National Statistics, at www.geoportal.statistics.gov.uk. LSOAs are standardized census and socio-demographic areas in England, designed to vary in geographic size but be relatively similar in population size. There are about 650 households [20] typically in each LSOA. The Index of Multiple Deprivation 2019 score (IMD2019) [21] for each LSOA was available from https://www.gov.uk/government/statistics/english-indices-of-deprivation-2019. The IMD2019 is a nationally calculated ranking of relative deprivation in LSOAs. These ranks were available in five ordinal categories (1 = most deprived; 5 = least deprived) relative to all-England. Similarly, the relative rurality of each respondent’s residence area was indicated by their LSOA in a taxonomy developed for the Department for the Environment, Food and Rural Affairs, available at www.geoportal.statistics.gov.uk [22]. These rurality designations were assigned based on many decision rules to put LSOAs into one of the four categories (from most urban to most rural, 1–4), depending on land use, population density and proximity to other high population density areas. All participant ages were divided into quintiles, to include age group as a potential confounder. Although all participants (cases or controls) were aged 70 at start of month when they were recruited, some had a birthday in the subsequent days right after invitation or their illness, and thus were of age 71 years when they filled in the questionnaire, just above age 70 years. We elected to retain these >70-year-old respondents in our study. Final age quintiles were hence 0–5, 6–18, 19–38, 39–56 and 57–71 years. Participant postcodes were also located in geographic region of England (9 possible).

### Analysis

Separate models were constructed for each individual STEC serotype for which at least 30 cases were detected and a combined group of all other STEC. Models were also generated that considered domestic-only risk factors, using only cases and controls that did not have a recent history of foreign travel. We did not consider specific domestic or foreign destinations as individual exposures; the destination counts were too heterogenous; a partial summary of just foreign destinations is provided in Supplementary Table S2. Models were constructed in Stata v.17. We did not model the effects of any exposure or risk factor if ≥95% of responses were identical, because apparent relationships could be skewed by small observation counts. Rather than impute missing data, where a datum on an included exposure was missing (such as age, occupation and other data that were not simple true/false answers in the questionnaires) that specific observation (case or control) was excluded in models using that same exposure. All correlates with *p* value < 0.20 in univariate analysis were trialed together in multivariate logistic regression to predict case status. Our preferred model was chosen by removing candidate correlates individually that had the highest *p* values until all correlates had *p* values≤0.20. We retained correlates with *p* value > 0.05 and <0.20 in the final models because it is plausible that these exposures might have a stronger association with case status if the dataset was much larger or these higher *p*-value exposures may act as important confounders. A kind of logistic regression was appropriate, given the binary nature of the outcome (case status or not). We opted against conditional regression because of imperfect matching of controls to cases, while mixed effects regression with all the candidate correlates proved unviable probably due to high complexity; these models could not converge to identify a best fit.

All models were adjusted by age quintile; there is evidence that specific STEC serotypes may have different age-distributions in cases [3]. However, the combined effects of the age-stratified control recruitment strategy and the age-divergent response rates (See the Results section) means that we cannot reliably treat age as a risk factor sampled without bias; so while all the models are adjusted for age quintile, we do not report coefficients on age quintiles. Multi-collinearity tests were undertaken to improve confidence in any associations found, using linear regression and variance inflation factor (vif) on the final logistic (multivariate) model dependent and independent variables.

Using adjusted ORs and the fraction of cases receiving an exposure, the population attributable risk percentage (PAR% [23] was determined for each factor that increased risk of being a case in the final models. The concept of population attributable risk or fraction is to estimate how much disease burden might be reduced if an exposure were eliminated.

## Results

### Description of study cohort

Each month, during the study timeframe of February 2019–March 2020, from 1 to 81 controls were recruited, while 23–101 cases were recorded in the UKHSA STEC surveillance system. The majority of cases were STEC O157 (*n* = 384/561, 68.4%) and STEC O26 (*n* = 50/561, 8.9%), while counts of other non-O157 serotypes (*n* = 127/561, 22.6%) were each below 30 and were not considered individually (Supplementary Table S3). STEC diagnoses peaked in August for all types of STEC, but seasonal peaks were more evident for O157 and O26 (Supplementary Figures S1a-1b). Return of control questionnaires peaked in June but were otherwise evenly spread throughout the months of the year. Ultimately, 561 cases and 600 controls questionnaires were included.

Median age of all STEC cases was 25 years. Overall response rate to control questionnaires was 5.45%. The controls response rate varied by age; the achieved age distribution of controls did not resemble age distribution of cases. Young adults (age 20–29) had the lowest response rates (1.87%) while adults aged 50–71 had the highest response rate (11.34%; Supplementary Table S1). As a result, the control population had fewer respondents in the age range 20–50 and more respondents aged 60–71 compared to any STEC case group (Supplementary Figures S2a-2d).

The proportion of females were similar for case and control groups, ranging from 52% to 60% (Table 1). IMD scores and rurality designations tended to be similar between groups, but age distribution and reports of recent foreign travel differed. Mann–Whitney U *p*-values for age distributions were statistically significantly different for each of group of O157, O26 and other-STEC cases compared to controls but not significantly different for O157 vs. other-STEC. Pearson’s test for proportionality (chi-square) between foreign travel exposure and any case and controls had a *p* value < 0.001 and was *p* = 0.066 between O157 and all non-O157 cases.

**Table 1.** Characteristics of participants in STEC case-control study performed in England, 2019–2020.

Trait	Controls *n* = 600	STEC O157 cases *n* = 384	STEC O26 cases *n*=50	Other STEC cases *n* = 127	All *n* = 1161
Female	56.0%	54.4%	52.0%	59.8%	55.7%
Median age (years)	33	26 **	15.5 **	26 **	27
Age IQR	8–59	10–46	2–31	7–50	8–52
age 60–71	30.7%	8.1% **	10.0% **	14.2% **	20.2%
Resident in most deprived quintile	11.9%	15.2%	8.0%	18.1% **	13.8%
Resident in a large urban area	32.9%	35.7%	38.0%	40.9% &	34.9%
History of recent foreign travel	7.5%	43.9% **	26% **	32.3% **	22.9%

Notes: For each type of case compared to controls, between group differences in medians tested with Pearson’s Chi-square test or Mann Whitney U test, and resulting levels of significant difference indicated as follows: & = *p* < 0.1; * = *p* < 0.05; ** = *p* < 0.01.

## Models

Univariate associations for exposures (as described in questionnaire, Supplementary) were similar for most exposures for O157, O26 and other STEC case status (Supplementary Table S4). The adjusted models are shown in two groups, with or without foreign travel considered as an exposure. We grouped models together in one table for brevity purposes and to make it easier for readers to compare relevance of the exposures to case status for each STEC group. These models are also adjusted for age, but coefficients are not shown due to age-targeting in recruitment methods. Other STEC = not O26 or O157 serotypes. Empty spaces in Tables 2 and 3 happen for correlates that were trialed in all of these adjusted models but only reached the retention threshold (*p* ≤ 0.20) for some of the serotype models. Supplementary Table S5 shows raw counts and percentages for each exposure described in the final adjusted models, summarized in Table 2.

**Table 2.** Multivariate models for exposures associated with STEC infections from England-based case-control study performed in 2019–2020.

	STEC O157, *n* = 935			STEC O26, *n* = 601			Other STEC, *n* = 679		
Factor	OR	95% CI	*P* > |z|	OR	95% CI	*P* > |z|	OR	95% CI	*P* > |z|
Childcare Occ’n	5.47	3.21–9.33	<0.001	11.16	3.84–32.39	<0.001	7.66	3.61–16.26	<0.001
Travel out of U.K.	8.81	5.61–13.82	<0.001	7.50	2.90–19.40	<0.001	8.78	4.52–17.08	<0.001
Travel in U.K.							0.56	0.30–1.06	0.076
Swam in Sea	2.18	1.22–3.90	0.009						
Dogs	0.74	0.53–1.03	0.072	0.51	0.24–1.08	0.077	0.57	0.34–0.95	0.033
Non dom an.							3.88	1.70–8.89	0.001
Day Trips	0.48	0.33–0.70	<0.001	0.38	0.17–0.87	0.021	0.18	0.09–0.37	<0.001
Petting Zoo				4.01	1.49–10.76	0.006			
Food At Zoo							0.29	0.09–1.00	0.050
Soil/Muck	0.40	0.27–0.60	<0.001	0.56	0.26–1.21	0.141	0.28	0.14–0.53	<0.001
*Season*									
Winter	1	(ref)	0.002	1	(ref)	0.087			
Spring	0.72	0.41–1.28		7.18	0.83–62.14				
Summer	1.69	1.01–2.82		11.40	1.39–93.57				
Autumn	1.53	0.90–2.59		5.78	0.69–48.76				
*Region*									
East Midlands	0.55	0.25–1.20	0.003	0.05	0.00–0.86	0.033	0.31	0.11–0.90	<0.001
East of England	0.39	0.20–0.75		0.24	0.05–1.06		0.10	0.04–0.30	
London	0.60	0.33–1.08		0.48	0.16–148		0.36	0.17–0.75	
North East	0.93	0.43–2.01		0.70	0.14–3.66		0.39	0.11–1.37	
North West	0.75	0.42–1.33		0.16	0.03–0.87		0.17	0.07–0.45	
South East	1	(ref)		1	(ref)		1	(ref)	
South West	1.20	0.66–2.18		1.13	0.37–3.44		0.19	0.07–0.52	
West Midlands	0.32	0.17–0.62		0.46	0.14–1.51		0.23	0.10–0.52	
Yorksh & Humber	0.94	0.52–1.69		0.10	0.02–0.54		0.16	0.06–0.42	
*Deprivation Quintile*									
1st (most deprived)				1	(ref)	0.052			
2nd				5.22	1.29–21.12				
3rd				3.33	0.82–13.59				
4th				1.29	0.29–5.77				
5th (least)				1.95	0.47–8.06				

Notes: Childc. Occ’n = Childcare occupation. Day Trips = trips to countryside or beach, asked about in addition to attendance at agricultural events or visits to zoo/petting zoo. ‘Swallowed water’ refers to while swimming. Soil/Muck = soil, sewage or manure. Non dom an. = Non- domesticated animals, which were livestock or wildlife, but not household pets such as cats or dogs. Significance denoted by fill & font colors: *p* < 0.05; *p* = 0.05–0.1, *p* = 0.1–0.19. *n* = # is count of combined cases and controls used to construct each model. Blank areas mean that a correlate was not retained (*p* values > 0.20) in the final adjusted model for that STEC group.

**Table 3.** Multivariate models for exposures for STEC infections (excluding respondents without history of foreign travel) from England-based case-control study performed in 2019–2020.

	STEC O157, *n* = 727			STEC O26, *n* = 547			Other STEC, *n* = 596		
Factor	OR	95% CI	*P* >|z|	OR	95% CI	*P* > |z|	OR	95% CI	*P* > |z|
Childcare Occ’n	5.67	3.26–9.84	<0.001	14.72	4.97–43.7	<0.001	8.68	3.74–20.15	<0.001
Swam in Sea	2.97	1.38–6.40	0.005						
Swallowed water	0.63	0.35–1.12	0.115						
Dogs				0.49	0.22–1.10	0.084	0.53	0.30–0.96	0.035
Non Dom an.							2.44	0.99–6.02	0.052
Petting Zoo				5.61	2.14–14.74	<0.001			
Day Trips	0.48	0.31–0.75	0.001	0.43	0.18–1.04	0.061	0.09	0.32–0.23	<0.001
Paddock/Field							2.06	1.04–5.43	0.039
Soil/Muck	0.47	0.31–0.72	0.001				0.30	0.14–0.62	0.001
*Season*									
Winter	1	(ref)	0.015						
Spring	0.77	0.40–1.47							
Summer	1.53	0.86–2.73							
Autumn	1.67	0.93–3.00							
*Region*									
East Midlands	0.75	0.32–1.77	0.010				0.54	0.18–1.60	<0.001
East of England	0.38	0.18–0.79					0.05	0.01–0.25	
London	0.69	0.36–1.33					0.41	0.18–0.94	
North East	0.80	0.34–1.89					0.41	0.11–1.51	
North West	0.77	0.41–1.46					0.15	0.05–0.44	
South East	1	(ref)					1	(ref)	
South West	1.25	0.66–2.37					0.22	0.08–0.64	
West Midlands	0.34	0.16–0.72					0.24	0.10–0.60	
Yorksh & Humber	1.23	0.65–2.32					0.32	0.12–0.86	
*Deprivation Quintile*									
1st (most deprived)				1	(ref)	0.054			
2^nd^				4.65	1.11–19.5				
3^rd^				1.37	0.32–5.95				
4^th^				1.5	0.31–7.17				
5th (least)				1.22	0.28–5.29				

Notes: Childc. Occ’n = Childcare occupation. Day Trips = trips to countryside or beach, asked about in addition to attendance at agricultural events or visits to zoo/petting zoo. ‘Swallowed water’ refers to while swimming. Soil/Muck = soil, sewage or manure. Non dom an. = Non- domesticated animals, which were livestock or wildlife, but not household pets such as cats or dogs. Significance denoted by fill & font colors: *p* < 0.05; *p* = 0.05–0.1, *p* = 0.1–0.19. *n* = # is count of combined cases and controls used to construct each model. Blank areas mean that a correlate was not retained (*p* values > 0.20) in the final adjusted model for that STEC group.

In the all-exposures models (Table 2), travel outside the U.K. (OR 5.47–11.16, all *p* < 0.001) and childcare occupation (OR 7.50–8.81, all *p* < 0.001) increased the risk of being any type of case. Exposure to dogs (OR 0.51–0.74) was recurringly protective (*p* always<0.10). Day trips were linked to lower case risk for all types of STEC (*p*  < 0.05 always). Exposure to soil or manure/sewage) was linked to lower risk for O157 and other STEC (at *p* < 0.001) and suggestive of reducing risk or confounding for O26 (OR 0.56, *p* = 0.141). Swimming in the sea had a higher risk for O157 (OR 2.18, *p* = 0.009) while becoming infected during the summer suggested higher risk for STEC O157 (OR 1.69, *p* = 0.002) or O26 (OR 11.40, *p* = 0.087) infections but not other STEC. Petting zoo visits were linked to possibly higher risk of having O26 (OR 4.01, *p* = 0.006) but not O157 or other STEC. Deprivation was associated with STEC O26 infection (*p* = 0.052) but not the other types of STEC. For other STEC but not O26 and O157, exposure to non-domesticated animals raised risk (OR 3.88, *p* = 0.001), travel in U.K. was linked to lower likelihood of case status (OR 0.56, *p* = 0.076) and having food at a zoo or agricultural event was associated with lower risk (OR 0.29, *p* = 0.050).

For cases and controls without a history of foreign travel, the relationships between exposures and association with often similar ones (comparing Tables 3 and 2). Examples of differences include ‘contact with dogs’ no longer being associated with lower risk of infection for O157 cases (at *p* ≤ 0.20), and travel within the U.K. not linked to case status for any type of STEC. In this subset of domestic-only cases and controls, seasonality was associated with O157 case status (peak OR was 1.67, for autumn, *p* = 0.015) but not case status for O26 or other STEC. Lower risk of being a case dependent on participant’s resident region emerged in most models, although resident region was not predictive domestic-only status with O26 infection (Table 3). A more comprehensive analysis of environmental exposures is planned separately for geospatial data linked to our STEC case and control data.

Collinearity tests (Supplementary Table S6) suggested low risk of multi-collinearity in models (all variance inflation factors were low, mostly below 2.0 and all <3.0). Foreign travel had the highest attributable risk for STEC O157, with PAR = 38.3% for O157 vs. 22.5% for O26 and 28.6% for other non-O157 (Table 4). The PAR% for childcare varied between 14.7% (O157 adjusted for foreign travel) and 25.2% (O26 model, domestic exposures only). Summer season was especially strongly associated with O26 (PAR = 43.8% in models that included foreign travel exposure). Swimming in the sea had lower PAR% among domestic cases than those in included foreign travel for O157 (PAR 5.8% vs. 11.7%), while the increased infection risk for O26 after petting zoo or agricultural fair visits was much stronger in the domestic exposures model than when adjusted for foreign travel (PAR = 24.4% vs. 18.0%).

**Table 4.** Population attributable risk percentages for exposures that increase the risk of being an STEC case in England in multivariate models.

With foreign travel					No foreign travel		
Factor	O157	O26	Other		O157	O26	Other
Travel out of U.K.	38.3%	22.5%	28.6%				
Childcare occupation	14.7%	21.8%	17.1%		18.9%	25.2%	19.5%
Swam in Sea	11.7%				5.8%		
Petting Zoo visit		18.0%				24.4%	
NonDom Animals			11.7%				8.9%
			Paddock/Field				10.2%
*Season*							
Summer	18.2%	43.8%			13.3%		
Autumn	9.9%				13.4%		
*Deprivation*							
2^nd^ quintile		25.9%				27.6%	
3^rd^ quintile							
4^th^ quintile							
5^th^ quintile							

Notes: PAR = Population attributable risk, from Rockill et al. (1998). NonDom Animals = contact with non-domesticated animals, paddock/field = spent time in this kind of place. Blank cells mean that factor did not (at *p* ≤ 0.05) raise risk for that type of infection relative to reference category in models shown in Tables 2–3.

## Discussion

Historically, epidemiological data linked to outbreaks of STEC have been used to identify animal reservoirs, high-risk food vehicles and high-risk environmental activities [24–27]. In England, a rich potential source of epidemiological data routinely collected for sporadic cases is an ESQ that is administered to every case of STEC O157 and a proportion of non-O157 STEC cases [3,8,28]. Using a case control study, we analyzed this collected data to identify animal and environmental risk factors associated with sporadic STEC infection in England.

Overall, the case and control groups exhibited similar characteristics with respect to male:female ratios, IMD scores and rurality designation; however on average the participants in the control group were older than cases and reported significantly less travel outside the U.K.. The association of STEC O157 infection with young children is well established in England [2,8], and there is evidence that other STEC serotypes that have the potential to cause HUS (specifically O26:H11, O145:H28, O80:H2) are also more common in children [9,29]. Childcare occupations often involve giving personal care (help with toileting, eating and dressing), activities which increase the risk of person-to-person transmission of a STEC pathogen. Previous analysis of routine surveillance data showed that 20–30% of STEC O157 infections were travel associated [2,8]. A separate analysis of non-O157 STEC travel data indicated that certain serotypes (e.g. STEC O117:H7) are more commonly associated with travel than others [3].

Previous studies concluded that the incidence of STEC O157 peaked in the summer months and the reasons for this are likely to be multifactorial [8]. Ruminants are the main zoonotic reservoir for STEC, and cattle and sheep are put out to graze in the U.K. from early spring to late autumn, thus increasing opportunities for direct contact with animals and/or their contaminated environment [30,31]. During the summer months, people are more likely to spend time in rural environments, and there is some evidence that they may be more at risk of eating contaminated produce and under-cooked barbequed meat. O26 and other STEC cases did not exhibit the same seasonal patterns as STEC O157, suggesting different animal reservoirs and transmission routes. Cases of STEC O26 exhibited similar seasonal patterns as STEC O157 only when travel-associated cases were included suggesting that travel outside the U.K. may be an important factor contributing to the summer peak of STEC O26 diagnoses in U.K. residents.

With regards to swimming in the sea, we note this effect was separate from seasonality. It may reflect localized, bathing water quality control issues [32], although swimming did not emerge as significant in the multivariate models for O26 or other non-O157 STEC. Swimming in the sea was reported year-round but it was ambiguous in the ESQ question and answers (hence also in the controls questionnaire) whether the sea swimming respondents were asked about was in U.K. or outside U.K. Similarly, the questions were phrased without specificity about whether pool or outdoor water was swallowed; these ambiguities underscore the challenges when trying to use an ESQ template for a case control study.

The last STEC case-control study performed in England was prospective and unmatched, undertaken in 1996–1997 and relied on self-administered questionnaires sent in by patients and controls from same primary care catchment areas (response rates, respectively, 84% and 57%) [13]. Cases with a history of foreign travel in the 5 days before illness onset were excluded. O’Brien *et al*. found that many types of farm/outdoor animal contact (for non-farm workers), paddling/wading and travel (nights away from home within the U.K.) were associated with acquiring STEC O157 infections, with odds ratios between 2.13 and 2.45. ‘Swimming in the sea’ in our study and the ‘paddling/wading’ exposure in O’Brien *et al*. demonstrated a similar risk in both studies.

Historically, petting farms have been recognized as a high-risk environmental exposure for STEC O157 in England [33]. This study provides evidence that this setting is also a risk for STEC O26 infection. Higher deprivation was only linked to O26 cases, for unclear reasons. Previous studies have associated cases of STEC O157 more commonly with affluent areas [34]. A rationale proposed for this was that people living in more deprived areas have higher chronic exposure and therefore levels of immunity [35]. Because STEC O26 is an emerging serotype, levels of immunity to O26 in deprived populations may still be low.

Overall, we found that non-food related risk or protective factors for STEC disease from O157 or non-O157 *E.coli* are broadly similar in magnitude and direction. It was surprising that contact with dogs, visits to outdoor spaces and contact with soil or muck decreased the risk of STEC infection. Dogs are known to be transient carriers of STEC [36] and sampling of outdoor spaces including surfaces of unpaved footpaths indicate that STEC bacteria are widespread in these settings [37]. It is possible that people with dogs and/or who have frequent exposure to mud and muck may have built up higher resistance to STEC illness.

### Strengths and limitations

A chief strength of our study is that we identified possibly different risk factors for developing O157 and non-O157 STEC disease, including a novel analysis of the O26 serotype. Data were collected concurrently from cases and controls using structured questionnaires. However, our response rate was low, especially for young adults. We do not know if measurement biases were introduced because of the different data collection methods (telephone interviews for cases and self-administered questionnaires for controls). The demographic differences our study achieved between the controls (who tended to be older adults) and cases may have resulted in some exposures appearing to be important or insignificant with regard to infection, when actually these statistical differences were due to the controls not being adequately similar to cases. This potential problem might be best addressed by sensitivity analysis, ideally using a much larger sample of controls than we had. Challenges in obtaining adequately similar controls also support a case for undertaking future case-control studies, especially if those can consider diverse STEC variants, to look for consistency in apparent relevance of candidate exposures. Exclusion of cases and controls above 70 years limits our information about infection in older adults. We acknowledge that the lack of food-related exposures in the model is also undesirable. The exercise highlights the difficulties of using public surveillance data to undertake a case-control study.

The existence of the Enhanced Surveillance for STEC database provided a potentially rich dataset for a case control study, but we found that some of the exposure data (food-related) were unsuitable. The ESQ is probably well designed to help identify food-borne disease in specific outbreak settings, but not with regard to identifying relative importance of broad food exposure categories. We have more confidence analyzing the other exposure data collected by the questionnaires (e.g. for traits related to residence area as denoted by home postcode, swimming history, travel history, etc.) because those types of information were observably not heavily biased by case status, yet were likely to be fixed over short periods (e.g. residence, age), not asked about in possibly different contexts and/or not open to multiple interpretations in how to answer.

Consideration of infection risk factors for non-O157 STEC infection has been made possible because of recent improvements in serotyping and surveillance. Separating analysis of epidemiological data linked to non-O157 STEC cases is desirable because emerging evidence indicates that each serotype has different levels of association with diverse animal reservoirs and a wide variety of transmission routes. Since this study was conducted in 2019–2020, there has been continued increase in the incidence of clinical cases of non-O157 STEC in England (C. Jenkins, pers. comm.). We recommend further case-control studies using epidemiological data linked to individual STEC serotypes to provide evidence of the associated animal reservoirs, food vehicles and environmental exposures. Such studies will provide an evidence base that will facilitate outbreak investigations and the implementation of public health interventions and inform public health guidance and policy.

## Supplementary Material

Supplemental Material
