# Supplementary material for: Animal and environmental risk factors for sporadic Shiga toxin-producing Escherichia coli (STEC) infection in England: a case control study for O157, O26 and other STEC serotypes
Source: Pathog Glob Health. 2023 Apr 4;117(7):655–63. doi: 10.1080/20477724.2023.2197672 (PMC10498794; doi:10.1080/20477724.2023.2197672)
Supplement: Supplemental Material [file YPGH_A_2197672_SM5976.zip › Appendix 1 rev2 (1).docx]

**APPENDIX 1**

**Materials and Methods (Additional Information)**

Handling Food Exposure Data from Questionnaires

Although both questionnaires asked about meals away from home, food preparation (eg., handling raw vegetables) and food ingestion (eg., cooked chicken, raw fruit, ice cream etc.) we found that food-related reporting was highly inconsistent and ultimately unreliable. For instance, both controls and cases sometimes reported eating specific food items at venues outside the home (eg., “chicken nuggets”) but later stated they had not eaten the same food category (eg., “cooked chicken”). The same problem was observed for many respondents with regard to many types of food, such as salad items, beef, cheeses, etc. We suspect that when specific foods were asked about after the section about eating outside the home., many respondents felt they should only report additional information. However, *some* respondents did include their restaurant meal ingestions in category exposures, stating the same meal and venue details repeatedly. We also note that respondents didn’t categorise their food intake consistently; for instance, some included ham as ‘cooked pork’ but not as ‘cured meat’. To try to remedy these inconsistencies would have been a large cleaning exercise for all (>1100) case and control answers.

Furthermore, in the week prior to infection, 114 STEC O157 cases and 41 STEC non-O157 cases (respectively 30% and 23% of each type of case) reported consuming none of the food items on the ESQ. It was implausible that all of these individuals had such limited diets as to eat no animal product, raw fruit or vegetables in this period. The same respondents also reported no food preparation or meals outside the home. There was not a similarly high level of entirely missing information for food-related questions among controls. We could not impute food-linked data for cases because imputation for > 20% of case observations would be undesirable (1) *and* we lacked confidence about which were missing answers or meant true absence of consumption. We considered many approaches to try to include the food-related data in spite of these inconsistencies and omissions but ultimately decided that the food reporting problems were artefacts of how the questionnaires were written and/or administered, and that our study results would be made much more reliable by only considering non-food risk factors. We feel confident that the non-food exposures and factors that the questionnaire asked about are more reliably reported than food exposures because they are more consistent conditions (such as occupation, age, domestic animal contact, residence) or more distinctive experiences (such as travel, wild animal contact or day trips).

**Table 1. Response rate (control questionnaires)** by original target age stratification group

| **AGE RANGES** | **Known**  **O157**  **case %s** | **Control Qnrs Sent out** | **Received** | **Proportion achieved (where age known)** | **Age-specific Response rate** |
| --- | --- | --- | --- | --- | --- |
| 0 - 4 | 20.91% | 2300 | 91 | 16.1% | 3.96% |
| 5 - 9 | 10.75% | 1182 | 81 | 14.4% | 6.85% |
| 10 - 19 | 12.68% | 1395 | 63 | 11.2% | 4.52% |
| 20 - 29 | 18.97% | 2087 | 39 | 6.9% | 1.87% |
| 30 - 49 | 19.94% | 2193 | 81 | 14.4% | 3.69% |
| 50 - 70 | 16.75% | 1843 | 209 | 37.1% | 11.34% |

*Note*: Qnrs = Questionnaires. Historically known case numbers were identified from (2).

**Table 2: Summary of foreign travel destinations**

| **Destination (country)** | **Controls** | **All cases** | **O157** | **Other STEC** |
| --- | --- | --- | --- | --- |
| *Nowhere/Blank* | 553 | 338 | 215 | 123 |
| Albania |  | 1 | 1 |  |
| Antigua |  | 1 | 1 |  |
| Australia | 2 | 3 | 3 |  |
| Austria | 2 |  |  |  |
| Azerbaijan |  | 1 | 1 |  |
| Bangladesh | 1 | 2 | 2 |  |
| Belgium |  | 3 | 2 | 1 |
| Bulgaria | 2 | 4 | 2 | 2 |
| China | 1 | 1 | 1 |  |
| Croatia |  | 3 | 2 | 1 |
| Cyprus | 2 | 9 | 9 |  |
| Czech Rep | 1 |  |  |  |
| Denmark | 1 | 1 | 1 |  |
| Egypt |  | 22 | 13 | 9 |
| Finland |  | 1 | 1 |  |
| France | 3 | 11 | 6 | 5 |
| Germany | 2 | 3 | 1 | 2 |
| Greece | 3 | 9 | 8 | 1 |
| Hungary | 1 | 1 | 1 |  |
| India | 1 | 2 | 1 | 1 |
| Indonesia |  | 1 | 1 |  |
| Iran |  | 1 | 1 |  |
| Iraq | 1 |  |  |  |
| Irish Republic |  | 2 | 1 | 1 |
| Israel |  | 4 | 3 | 1 |
| Italy |  | 5 | 3 | 2 |
| Jamaica |  | 1 |  | 1 |
| Kenya |  | 1 |  | 1 |
| Malaysia |  | 1 | 1 |  |
| Malta |  | 4 | 3 | 1 |
| Mexico |  | 6 | 2 | 4 |
| Montenegro |  | 1 |  | 1 |
| Morocco |  | 3 | 3 |  |
| Netherlands |  | 2 | 2 |  |
| New Zealand | 1 |  |  |  |
| Nigeria |  | 1 | 1 |  |
| Norway | 1 |  |  |  |
| Oman |  | 1 | 1 |  |
| Pakistan | 1 | 1 | 1 |  |
| Poland | 4 | 2 | 1 | 1 |
| Portugal | 3 | 8 | 8 |  |
| Romania |  | 1 | 1 |  |
| Russia |  | 1 |  | 1 |
| Qatar | 1 |  |  |  |
| Slovakia |  | 1 | 1 |  |
| South Africa |  | 1 | 1 |  |
| South Korea |  | 1 |  | 1 |
| Spain | 9 | 18 | 13 | 5 |
| Switzerland |  | 2 | 1 | 1 |
| Tanzania | 1 |  |  |  |
| Thailand | 1 | 2 | 1 | 1 |
| Tunisia |  | 1 | 1 |  |
| Turkey | 3 | 43 | 36 | 7 |
| Uganda |  | 1 |  | 1 |
| United Arab Emirates |  | 6 | 6 |  |
| USA | 2 | 2 | 2 |  |
| Other Africa |  | 5 | 3 | 2 |
| Other Asia |  | 6 | 4 | 2 |
| Other Caribbean |  | 1 |  | 1 |
| Other Europe | 3 |  |  |  |
| Other Middle East | 1 |  |  |  |
| Other Central & South America | 1 | 1 |  | 1 |

*Notes*: Empty space = Zero persons mentioned this destination. Some respondents reported multiple foreign destinations and are counted twice or more often (in same column).

**Table 3. Frequency of non-O157 serotypes** that were included in the case control study.

| **Serotype** | **Count** |  | **Serotype** | **Count** |  | **Serotype** | **Count** |
| --- | --- | --- | --- | --- | --- | --- | --- |
| UNIDENTIFIABLE: | 3 |  | O146: | 18 |  | O38: | 1 |
| O103: | 8 |  | O146:H21 | 4 |  | O45: | 1 |
| O103:H2 | 2 |  | O146:H28 | 1 |  | O45:H2 | 2 |
| O104: | 1 |  | O151:H2 | 1 |  | O63:H6 | 1 |
| O111: | 4 |  | O153-O178: | 1 |  | O71:H8 | 2 |
| O113: | 3 |  | O156: | 3 |  | O78: | 2 |
| O116:H48 | 1 |  | O156:H25 | 1 |  | O8:H16 | 1 |
| O117: | 3 |  | O165: | 1 |  | O8:H19 | 1 |
| O117:H7 | 1 |  | O166: | 1 |  | O80: | 2 |
| O121:H19 | 1 |  | O166:H28 | 1 |  | O81:H21 | 1 |
| O123: | 3 |  | O174:H2 | 1 |  | O82: | 1 |
| O128AB: | 5 |  | O177: | 5 |  | O84: | 1 |
| O128ab:H2 | 3 |  | O181: | 1 |  | O85: | 1 |
| O13-O135: | 1 |  | O182: | 1 |  | O87: | 1 |
| O142: | 1 |  | O25:H4 | 1 |  | O9: | 1 |
| O145: | 11 |  | O26: | 45 |  | O91: | 9 |
| O145:H28 | 7 |  | O26:H11 | 5 |  |  |  |
|  |  |  |  |  |  | **Grand Total** | **177** |

*Note*: Colour-coding of same-serovar groups was used to make them stand out; the colours don’t indicate anything else.

**Figures 1a and 1b. Questionnaires received per month** (as counts or percentages of total).

Notes: 21 control questionnaires arrived in February 2020 and one in March 2020. Two control questionnaires were undated hence total = 598 not 600. Multi-year English or all-UK data may be worth analysing in future to determine if case detection of specific serotypes have different seasonal patterns, perhaps reflecting seasonal variations in underlying risk distribution exposures which were indicated in Irish analysis of 2015 data as reported in (3).

**Figures 2a-2d. Age distributions** of each type of case and controls

The age distributions were dissimilar between groups, although somewhat more similar between O26 and other types of non-O157 STEC. The charts also show how intended age-frequency matching between O157 cases and controls was imperfect.

| 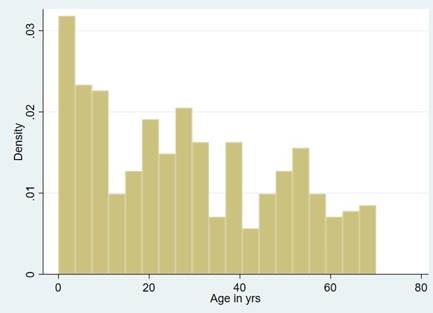  2a O157 cases | 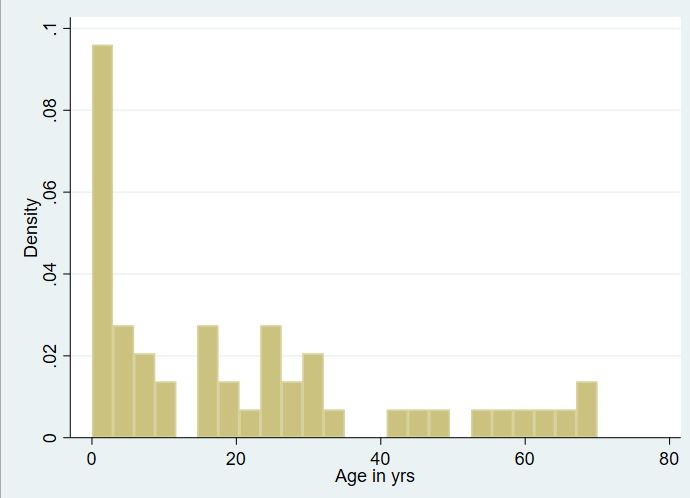  2b O26 cases |
| --- | --- |
| 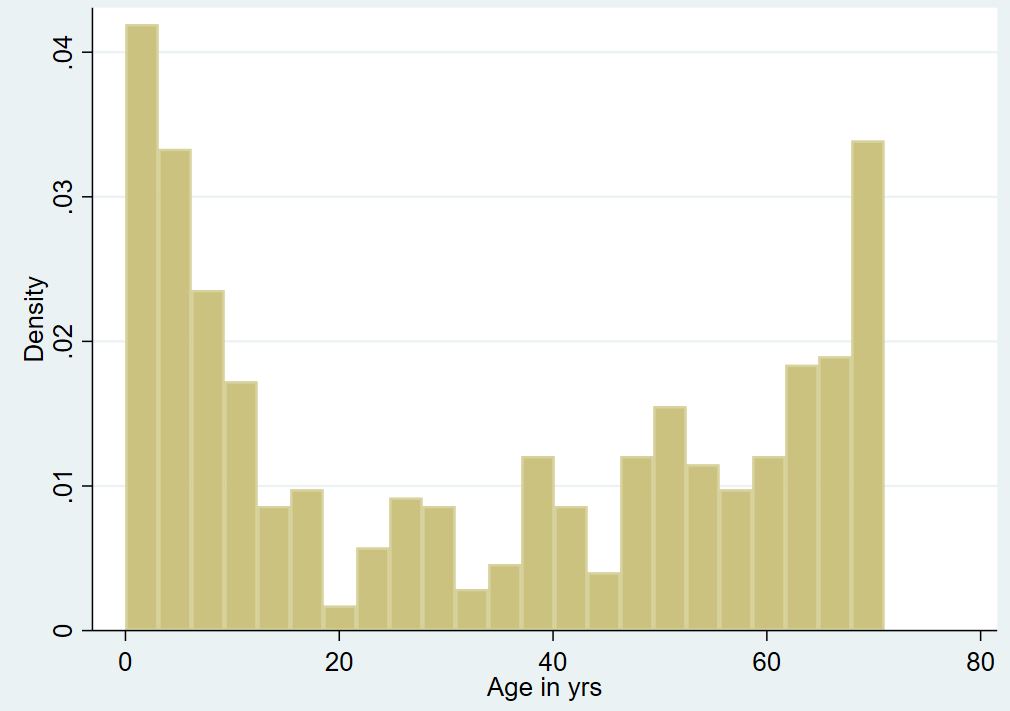  2c Controls | 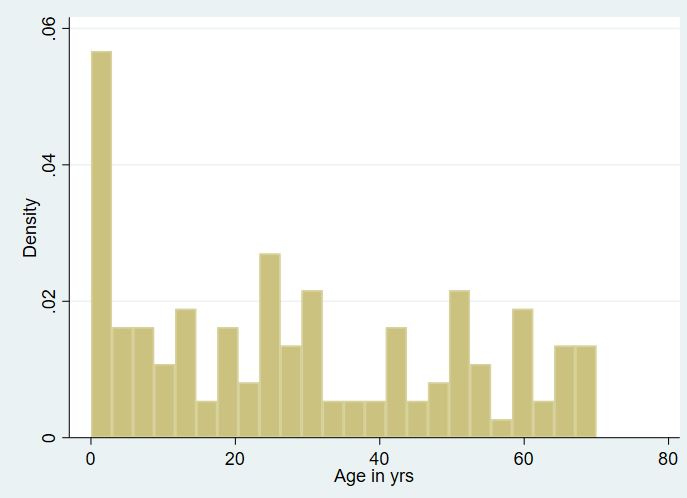  2d Other STEC cases |

*Note***:**  Counts are expressed as % of total dataset (“density”) on vertical axes, for age groups (horizontal axes).

**Table 4.** Univariate analysis for case status, O157, O26 or all other STEC

|  |  | **O157** |  |  | **O26** |  |  |  |  | **Other** |  |
| --- | --- | --- | --- | --- | --- | --- | --- | --- | --- | --- | --- |
| Factor | **OR** | **95% CI** | **P>\|z\|** |  | **OR** | **95% CI** | **P>\|z\|** |  | **OR** | **95% CI** | **P>\|z\|** |
| **Gender (1M,0F)** | 1.06 | 0.82-1.38 | 0.636 |  | 1.17 | 0.66-2.09 | 0.588 |  | 0.86 | 0.58-1.26 | 0.424 |
| **Children in Home** | 0.89 | 0.69-1.16 | 0.4 |  | 1.41 | 0.79-2.51 | 0.244 |  | 0.89 | 0.60-1.31 | 0.544 |
| **Childcare occupation** | 4.31 | 2.74-6.80 | < 0.001 |  | 6.22 | 2.94-13.14 | < 0.001 |  | 4.83 | 2.72-8.58 | < 0.001 |
| **Travel outside UK** | 9.39 | 6.52-13.53 | < 0.001 |  | 4.33 | 2.15-8.74 | < 0.001 |  | 5.88 | 3.64-9.50 | < 0.001 |
| **Travel in UK** | 0.70 | 0.52-0.95 | 0.02 |  | 0.96 | 0.51-1.82 | 0.9 |  | 0.46 | 0.28-0.77 | 0.003 |
| *Swimming history* |  |  |  |  |  |  |  |  |  |  |  |
| **in Fresh water** | 0.99 | 0.73-1.33 | 0.927 |  | 0.85 | 0.42-1.69 | 0.637 |  | 0.74 | 0.46-1.18 | 0.204 |
| **in Sea** | 3.66 | 2.46-5.45 | < 0.001 |  | 1.81 | 0.73-4.50 | 0.199 |  | 2.05 | 1.13-3.74 | 0.019 |
| **Swallowed water** | 1.28 | 0.92-1.78 | 0.146 |  | 0.71 | 0.29-1.71 | 0.44 |  | 0.75 | 0.42-1.32 | 0.216 |
| *Contact with animals* |  |  |  |  |  |  |  |  |  |  |  |
| **Any Animal** | 0.83 | 0.63-1.09 | 0.174 |  | 0.72 | 0.40-1.30 | 0.281 |  | 0.47 | 0.32-0.70 | < 0.001 |
| **Domestic Animal** | 0.76 | 0.58-0.99 | 0.043 |  | 0.77 | 0.43-1.39 | 0.392 |  | 0.42 | 0.28-0.62 | < 0.001 |
| **Dogs** | 0.63 | 0.49-0.82 | 0.001 |  | 0.55 | 0.30-1.01 | 0.054 |  | 0.42 | 0.28-0.64 | < 0.001 |
| **Cats** | 1.04 | 0.78-1.38 | 0.786 |  | 1.03 | 0.54-1.97 | 0.919 |  | 0.56 | 0.34-0.91 | 0.02 |
| **NonDom Animals** | 0.70 | 0.46-1.07 | 0.101 |  | 1.75 | 0.84-3.65 | 0.135 |  | 1.31 | 0.77-2.23 | 0.325 |
| *Away from home* |  |  |  |  |  |  |  |  |  |  |  |
| **Petting Zoo** | 1.07 | 0.70-1.61 | 0.764 |  | 2.74 | 1.36-5.52 | 0.005 |  | 1.08 | 0.58-1.99 | 0.817 |
| **Food at Zoo/Agric** | 1.00 | 0.62-1.61 | 0.991 |  | 1.92 | 0.82-4.49 | 0.135 |  | 0.48 | 0.18-1.24 | 0.129 |
| **Paddock/Field** | 0.57 | 0.41-0.80 | 0.001 |  | 1.04 | 0.53-2.04 | 0.915 |  | 0.65 | 0.39-1.08 | 0.096 |
| **Other Day Trips** | 0.66 | 0.50-0.87 | 0.003 |  | 0.62 | 0.33-1.17 | 0.138 |  | 0.26 | 0.15-0.44 | < 0.001 |
| **Soil/Muck** | 0.31 | 0.22-0.43 | 0.001 |  | 0.62 | 0.32-1.19 | 0.152 |  | 0.31 | 0.19-0.52 | < 0.001 |
| *Questionnaire completed* |  |  |  |  |  |  |  |  |  |  |  |
| **Winter: Dec-Feb** | 1 | (ref) | < 0.001 |  | 1 | (ref) | 0.049 |  | 1 | (ref) | 0.984 |
| **Spring: Mar-May** | 0.63 | 0.40-1.01 |  |  | 6.57 | 0.83-51.7 |  |  | 0.94 | 0.51-1.72 |  |
| **Summer: June-Aug** | 1.71 | 1.14-2.56 |  |  | 12 | 1.60-90.1 |  |  | 0.93 | 0.52-1.67 |  |
| **Autumn: Sep-Nov** | 1.20 | 0.79-1.83 |  |  | 7.7 | 0.99-59.2 |  |  | 0.89 | 0.49-1.62 |  |
| *Deprivation residence area* |  |  |  |  |  |  |  |  |  |  |  |
| **IMD quintile 1** | 1 | (ref) | 0.12 |  | 1 | (ref) | 0.093 |  | 1 | (ref) | 0.066 |
| **IMD quintile 2** | 0.63 | 0.39-1.00 |  |  | 2.83 | 0.91-8.82 |  |  | 0.86 | 0.46-1.62 |  |
| **IMD quintile 3** | 0.77 | 0.50-1.20 |  |  | 1.64 | 0.51-5.28 |  |  | 0.45 | 0.23-0.89 |  |
| **IMD quintile 4** | 0.86 | 0.55-1.32 |  |  | 0.97 | 0.28-3.44 |  |  | 0.72 | 0.39-1.34 |  |
| **IMD quintile 5** | 0.61 | 0.40-0.94 |  |  | 1.17 | 0.36-3.81 |  |  | 0.5 | 0.27-0.93 |  |
| *Urban/rural residence* |  |  |  |  |  |  |  |  |  |  |  |
| **Conurbation** | 1 | (ref) | 0.26 |  | 1 | (ref) | 0.392 |  | 1 | (ref) | 0.228 |
| **Urban city/town** | 0.87 | 0.65-1.16 |  |  | 0.7 | 0.36-1.34 |  |  | 0.75 | 0.50-1.14 |  |
| **Rural towns** | 0.70 | 0.43-1.16 |  |  | 1.4 | 0.58-3.37 |  |  | 0.45 | 0.19-1.04 |  |
| **Villages, hamlets, very rural** | 1.22 | 0.75-1.98 |  |  | 0.68 | 0.19-2.39 |  |  | 0.74 | 0.34-1.62 |  |
| *Ethnicity* |  |  |  |  |  |  |  |  |  |  |  |
| **White** | 1 | (ref) | 0.55 |  | 1 | (ref) | 0.26 |  | 1 | (ref) | 0.083 |
| **Asian** | 0.65 | 0.30-1.40 |  |  | (empty) |  |  |  | 2.5 | 1.09-5.75 |  |
| **Black** | 0.69 | 0.27-1.76 |  |  | 1.08 | 0.14-8.42 |  |  | 0.99 | 0.22-4.40 |  |
| **Other** | 0.80 | 0.40-1.60 |  |  | 2.54 | 0.83-7.72 |  |  | 2.04 | 0.86-4.85 |  |
| *English Region* |  |  |  |  |  |  |  |  |  |  |  |
| **East Midlands** | 0.70 | 0.37-1.30 | 0.028 |  | 0.24 | 0.03-1.90 | 0.16 |  | 0.34 | 0.14-0.87 | < 0.001 |
| **East of England** | 0.53 | 0.32-0.87 |  |  | 0.32 | 0.09-1.18 |  |  | 0.15 | 0.06-0.37 |  |
| **London** | 0.83 | 0.52-1.32 |  |  | 1.16 | 0.47-2.82 |  |  | 0.64 | 0.36-1.14 |  |
| **North East** | 0.91 | 0.48-1.73 |  |  | 1.19 | 0.36-3.98 |  |  | 0.29 | 0.09-0.86 |  |
| **North West** | 0.73 | 0.46-1.17 |  |  | 0.22 | 0.05-1.02 |  |  | 0.19 | 0.08-0.43 |  |
| **South East** | 1 | (ref) |  |  | 1 | (ref) |  |  | 1 | (ref) |  |
| **South West** | 0.91 | 0.57-1.47 |  |  | 1.17 | 0.47-2.94 |  |  | 0.22 | 0.09-0.51 |  |
| **West Midlands** | 0.46 | 0.27-0.80 |  |  | 0.68 | 0.24-1.88 |  |  | 0.38 | 0.19-0.74 |  |
| **Yorkshire & Humber** | 1.14 | 0.71-1.81 |  |  | 0.41 | 0.11-1.51 |  |  | 0.33 | 0.15-0.69 |  |
|  |  |  |  |  |  |  |  |  |  |  |  |
| *Age Quintile (yrs)* |  |  |  |  |  |  |  |  |  |  |  |
| **0-5** | 1 | (ref) | < 0.001 |  | 1 | (ref) | 0.002 |  | 1 | (ref) | < 0.001 |
| **6 to 18** | 1.06 | 0.70-1.62 |  |  | 0.52 | 0.22-1.20 |  |  | 0.77 | 0.41-1.44 |  |
| **19 to 38** | 3.15 | 2.07-4.78 |  |  | 1.28 | 0.59-2.78 |  |  | 2.3 | 1.28-4.13 |  |
| **39-56** | 1.18 | 0.77-1.79 |  |  | 0.31 | 0.11-0.86 |  |  | 1.03 | 0.56-1.88 |  |
| **57-71** | 0.48 | 0.31-0.75 |  |  | 0.21 | 0.08-0.59 |  |  | 0.57 | 0.30-1.06 |  |

*Notes***:** Muck = manure or sewage. Significance denoted by fill & font colours: p < 0.05 ; p = 0.05-0.1; p = 0.1-0.19. Swallowed-water means yes they swallowed water while swimming. The IMD (Index of Multiple Deprivation) quintiles are as follows: (1 = most deprived to 5 = least deprived).

**Table 5. Raw counts (n) and percentages (%) of each type of case or controls with each exposure shown in Table 2 (adjusted model) in main article.**

| **Factor** | **Controls** | **Other STEC** | **STEC O26** | **STEC O157** |
| --- | --- | --- | --- | --- |
| **Childcare Occ’n** | 29, 4.8% | 25, 19.7% | 12, 24% | 69, 18.0% |
| **Travel out of UK** | 45, 7.5% | 41, 32.3% | 13, 26% | 166, 43.2% |
| **Travel in UK** | 173, 28.8% | 20, 15.7% | 14, 28% | 85, 22.1% |
| **Swam in Sea** | 42, 7.0% | 17, 13.4% | 6, 12.0% | 83, 21.6% |
| **Dogs** | 290, 48.3% | 36, 28.3% | 17, 34% | 143, 37.2% |
| **Non dom an.** | 75, 12.5% | 20, 15.7% | 10, 20.0% | 35, 9.1% |
| **Day Trips** | 232, 38.7% | 18, 14.2% | 14, 28.0% | 113, 29.4% |
| **Petting Zoo** | 62, 10.3% | 14, 11.0% | 12, 24.0% | 42, 10.9% |
| **Food At Zoo** | 47, 7.8% | 5, 3.9% | 7, 14.0% | 30, 7.8% |
| **Soil/Muck** | 217, 36.2% | 19, 15.0% | 13, 26.0% | 57, 14.8% |
| *Season* |  |  |  |  |
| **Winter** | 92, 15.4% | 21, 16.5% | 1, 2.0% | 50, 13.0% |
| **Spring** | 154, 25.7% | 33, 26.0% | 11, 22.0% | 53, 13.8% |
| **Summer** | 184, 30.8% | 39, 30.7% | 24, 48.0% | 171, 44.5% |
| **Autumn** | 168, 28.1% | 34, 26.8% | 14, 28.0% | 110, 28.6% |
| *Region* |  |  |  |  |
| **East Midlands** | 35, 6.0% | 6, 4.7% | 1, 2.0% | 20, 5.2% |
| **East of England** | 78, 13.3% | 6, 4.7% | 3, 6.0% | 34, 8.8% |
| **London** | 72, 12.3% | 23, 18.1% | 10, 20.0% | 49, 12.8% |
| **North East** | 28, 4.8% | 4, 3.1% | 4, 8.0% | 21, 5.5% |
| **North West** | 75, 12.8% | 7, 5.5% | 2, 4.0% | 45, 11.7% |
| **South East** | 100, 17.0% | 50, 39.4% | 12, 24.0% | 82, 21.3% |
| **South West** | 65, 10.9% | 7, 5.5% | 9, 18.0% | 48, 12.5% |
| **West Midlands** | 74, 12.6% | 14, 11.0% | 6, 12.0% | 28, 7.3% |
| **Yorksh & Humber** | 61, 10.4% | 10, 7.9% | 3, 6.0% | 57, 14.8% |
| *Deprivation Quintile* | |  |  |  |
| **1^st^, most deprived** | 70, 11.9% | 23, 18.1% | 4, 8.0% | 61, 16.0% |
| **2nd** | 99, 16.9% | 28, 22.0% | 16, 32.0% | 54, 14.1% |
| **3rd** | 128, 21.8% | 19, 15.0% | 12, 24.0% | 86, 22.5% |
| **4th** | 126, 21.5% | 30, 23.6% | 7, 14.0% | 94, 24.6% |
| **5^th^, least** | 164, 27.9% | 27, 21.3% | 11, 22.0% | 87, 22.8% |

**Table 6. Variance inflation factors for models of Table 2 and Table 3 in main article**

|  | *With foreign travel, Table 2* | | |  | *Without foreign travel, Table 3* | | |
| --- | --- | --- | --- | --- | --- | --- | --- |
|  | **O157** | **O26** | **Other** |  | **O157** | **O26** | **Other** |
| **Travel outside UK** | 1.25 | 1.05 | 1.06 |  |  |  |  |
| **Travel in UK** |  |  | 1.09 |  |  |  |  |
| **Childcare Occupation** | 1.03 | 1.06 | 1.03 |  | 1.04 | 1.05 | 1.04 |
| **Swam in Sea** | 1.28 |  |  |  | 1.16 |  |  |
| **Swallowed water** |  |  |  |  | 1.19 |  |  |
| **Day Trips** | 1.15 | 1.15 | 1.18 |  | 1.16 | 1.09 | 1.21 |
| **Dogs** | 1.05 | 1.08 | 1.09 |  |  | 1.04 | 1.09 |
| **Soil/Muck** | 1.13 | 1.13 | 1.17 |  | 1.10 |  | 1.17 |
| **Petting Zoo** |  | 1.17 |  |  |  | 1.14 |  |
| **Paddock/Field** |  |  |  |  |  |  | 1.32 |
| **NonDom Animals** |  |  | 1.28 |  |  |  | 1.19 |
| **Food at Zoo** |  |  | 1.24 |  |  |  |  |
| *Season* |  |  |  |  |  |  |  |
| **Winter** | na (ref) | na (ref) |  |  | na (ref) | na (ref) | na (ref) |
| **Spring** | 1.96 | 2.13 |  |  | 1.98 |  |  |
| **Summer** | 2.33 | 2.32 |  |  | 2.26 |  |  |
| **Autumn** | 2.18 | 2.18 |  |  | 2.18 |  |  |
| *Deprivation* |  |  |  |  |  |  |  |
| **1^st^ quintile (most)** |  | na (ref) |  |  |  | na (ref) | na (ref) |
| **2^nd^** |  | 2.18 |  |  |  | 2.16 |  |
| **3^rd^** |  | 2.44 |  |  |  | 2.45 |  |
| **4^th^** |  | 2.29 |  |  |  | 2.32 |  |
| **5^th^ (least deprived)** |  | 2.57 |  |  |  | 2.60 |  |
| *English Region* |  |  |  |  |  |  |  |
| **East Midlands** | 1.25 | 1.29 | 1.24 |  | 1.26 |  | 1.24 |
| **East of England** | 1.45 | 1.54 | 1.40 |  | 1.45 |  | 1.42 |
| **London** | 1.50 | 1.63 | 1.45 |  | 1.50 |  | 1.47 |
| **North East** | 1.22 | 1.28 | 1.19 |  | 1.23 |  | 1.21 |
| **North West** | 1.46 | 1.51 | 1.38 |  | 1.49 |  | 1.42 |
| **South East** | na (ref) | na (ref) | na (ref) |  | na (ref) |  | na (ref) |
| **South West** | 1.44 | 1.51 | 1.34 |  | 1.48 |  | 1.37 |
| **West Midlands** | 1.42 | 1.57 | 1.42 |  | 1.46 |  | 1.45 |
| **Yorkshire & Humber** | 1.46 | 1.47 | 1.33 |  | 1.44 |  | 1.33 |

*Note*: Soil/Muck = soil, manure or sewage; NonDom = non-domesticated animals. Blank spaces occur because that exposure was not retained in the final model.

## References for Appendix 1

1. Royston P. Multiple imputation of missing vaues. The Stata Journal. 2004;4(3):227-41.

2. England PH. Vero cytotoxin-producing Escherichia coli (VTEC) O157 data 2006 to 2015. 2016.

3. Garvey P, Carroll A, McNamara E, Charlett A, Danis K, McKeown PJ. Serogroup-specific seasonality of verotoxigenic Escherichia coli, Ireland. Emerging Infectious Diseases. 2016;22(4):742.
