# Supplementary material for: Animal and environmental risk factors for sporadic Shiga toxin-producing Escherichia coli (STEC) infection in England: a case control study for O157, O26 and other STEC serotypes
Source: Pathog Glob Health. 2023 Apr 4;117(7):655–63. doi: 10.1080/20477724.2023.2197672 (PMC10498794; doi:10.1080/20477724.2023.2197672)
Supplement: Supplemental Material [file YPGH_A_2197672_SM5976.zip › Appendix 2 Questionnaire.doc]

**Appendix #2**

**Adult Healthy Participant Questionnaire**

Personal Details

| **Unique Identifying Number (UIN):** | | | | | | |  | | | | | | |
| --- | --- | --- | --- | --- | --- | --- | --- | --- | --- | --- | --- | --- | --- |
| *This can be found on the invitation letter.* | | | | | | | | |  | | | | |
| **Date completed:** | | | | | / / | | | | | | | | |
|  | | | | | | | | |  | | | | |
| Postcode: | | | |  | | | | | | | | | |
| Sex: | M | | F | | | Age: yrs | | | | | | | |
| Are there any children living in the household? Y N Unknown | | | | | | | | | | | | | |
| Occupation: | |  | | | | | | | | | | Tick if any of the below risk groups apply | |
|  | | Food handler (e.g. handle food professionally) | | | | | | | | | | Work in/attend healthcare setting | |
|  |  | Work in/attend childcare setting | | | | | | | | | | Work in contact with faeces (e.g. lab, farm etc.) | |
|  |  | Have difficulty maintaining personal hygiene | | | | | | | | | | Other risk category | |
|  |  | If yes to any of the above, details: | | | | | | | | | | | |
| Ethnicity: | | White | | | | | | Mixed | | | Asian/Asian British | | Black/Black British |
|  | | Chinese | | | | | | Other: | |  | | | |
|  | |  | | | | | |  | |  | | | |

Your Travel in the last week

| **Travelled OUTSIDE of the UK:** | | | Y | | | N | | Not sure |
| --- | --- | --- | --- | --- | --- | --- | --- | --- |
| Specify countries visited (from most recent to least recent) | | | | | | | | |
| **Country/Region** | **Length of trip in days** | | | Type of accommodation used: | | | | Friends/Family |
|  |  | | | Hotel | | | | Tourist Campsite |
|  |  | | | Bed & Breakfast | | | | Holiday dwelling |
|  |  | | | Guesthouse | | | | Other (specify below) |
|  | | | |  | | | | |
|  | | |  | | |  | |  |
|  | | |  | | |  | |  |
| **Travelled WITHIN the UK:** | | | Y | | | N | | Not sure |
| Specify town/resort visited (from most recent to least recent) | | | | | | | | |
| **Town/Resort** | **Length of trip in days** | | | Type of accommodation used: | | | | Friends/Family |
|  |  | | | Hotel | | | | Tourist Campsite |
|  |  | | | Bed & Breakfast | | | | Holiday dwelling |
|  |  | | | Guesthouse | | | | Other (specify below) |
|  | | | | | | |  | |
|  | |  | | |  | | | |

Water exposure in the last week

| Did you drink unboiled water from any of the following water supplies?: | | | | | | | | Other water exposures: | | | | | |
| --- | --- | --- | --- | --- | --- | --- | --- | --- | --- | --- | --- | --- | --- |
|  |  | | | | **Yes** | | **No** |  | | | **Yes** | **No** | **Not sure** |
|  | Mains (municipal) water | | | |  | |  | Exposed to Floodwater | | |  |  |  |
| Private water supply (spring/well/borehole) | | | | |  | |  | Experienced any household | | |  |  |  |
|  | Bottled water | | | |  | |  | drainage/plumbing problems | | |  | | |
|  | Unboiled river/stream/lake water | | | |  | |  |  | | | | | |
|  | | | | | | | |  | | | | | |
|  | | | | | | | | | | | | | |
| Participated in any of the following activities – either recreationally or for work: | | | | | | | | | | | | | |
| **Activity** | | **Fresh water** | **Sea Water** | **No** | | **Details** | | | | | | | |
| Swimming/paddling (including swimming pools) | |  |  |  | |  | | | | | | | |
| Other (e.g. canoeing, fishing, sailing, surfing) | |  |  |  | |  | | | | | | | |
| Is it possible that water was accidentally swallowed during any of the above: | | | | | | | | | Y | N | | Not sure | |
|  | |  | | | | | | | | | | | |

Animal Contact in the LAST week

| Was there any contact with domestic animals/pets? | | | | | | | | | | | Y | | | | | | | N | | | |  | | | | | | | | | | | | | | |  | |
| --- | --- | --- | --- | --- | --- | --- | --- | --- | --- | --- | --- | --- | --- | --- | --- | --- | --- | --- | --- | --- | --- | --- | --- | --- | --- | --- | --- | --- | --- | --- | --- | --- | --- | --- | --- | --- | --- | --- |
| If yes, indicate which animals below: | | | | | | | |  | | | | | | | | | |  | | | |  | | | | | | | | | | | | | | |  | |
| Dogs | Cats | | | | | Rabbits | | | | | | Reptiles | | | | | | | | | | | Birds | | | | | | | | Fish | | | | | |  | |
| Other: |  | | | | |  | | | | | |  | | | | | | | | | | |  | | | | | | | |  | | | | | |  | |
| Did any of the above animals have diarrhoea: | | | | | | | | | | | Y | | | | | | N | | | | Not sure | | | | | | | | | | | | | | | | | |
| If yes, specify: | |  | | | | | | | | | | | | | | | | | | | | | | | | | | | | | | | | | | | | |
| Was there a veterinary investigation? | | | | | | | | | | | Y | | | | | N | | | | | Not sure | | | | | | | | | | | | | | | | | |
| Was there any contact with non domestic animals? | | | | | | | | | | | Y | | | | | | N | | | |  | | | | | | | | | | | | | | | | | |
| If yes, indicate which animals below: | | | | | | | | | | |  | | | | | |  | | | |  | | | | | | | | | | | | | | | | | |
| Cattle | | Calves | | | Sheep | | | | | Lambs | | | | | | | | | | Goats | | | | | Horses | | | | | | | | Pigs | | | | | |
| Poultry | | Rabbits | | | Deer | | | | | Birds | | | | | | | | | | Fish | | | | | Rodents | | | | | | | | Reptiles | | | | | |
| Other: |  | |  | | | | |  | | | | | | |  | | | | | | | | |  | | |  | | | | | | | |  | | | |
| Do you live on, work at, or have access to a private farm? | | | | | | | | | | | | | | Y | | | | | N | | | | | Not sure | |  | | | | | |  | | | |  | | |
| Did you attend an agricultural event e.g. horse show? | | | | | | | | | | | | | | Y | | | | | N | | | | | Not sure | |  | | | | | |  | | | |  | | |
| Did you visit a farm/petting zoo/bird reserve, etc? | | | | | | | | | | | | | | Y | | | | | N | | | | | Not sure | | | | |  | | |  | | | |  | | |
| If yes, did you handle the animals?: | | | | | | | | | Y | | | | N | | | | | | Not sure | | | | | |  | | | | | | | | | | | | |  |
| Did you bottle feed any of the animals?: | | | | | | | | | Y | | | | N | | | | | | Not sure | | | | | | | | | | | | | | | | | | |  |
| If yes to any of above, did you wash your hands before eating food or before leaving?: | | | | | | | | | | | | | | | | | | | | | | | | | Y | | | | | N | | | Not sure | | | | | |
|  | | | | | | | | |  | | | |  | | | | | |  | | | | | | | | | | | | | | | | | |  | |
|  | | | | | | | | |  | | | |  | | | | | |  | | | | | | | | | | | | | | | | | |  | |
| Did you consume any food whilst there?: | | | | | | | | | Y | | | | N | | | | | | Not sure | | | | | | | | | | | | | | | | | |  | |
| Was the food: | | | | | | |  | | | | | | | | | | | | | | | | | | | | |  | | | | | |  | | |  | |
| Purchased on premises | | | | OR | | | | Brought from home/elsewhere | | | | | | | | | | | | | | | | | | | | | | | | | | | | |  | |
| Eaten in a separate area | | | | OR | | | | Eaten whilst in contact with the animals | | | | | | | | | | | | | | | | | | | | | | | | | | | | |  | |
| Eaten at a picnic table | | | | OR | | | | Eaten while sat on grass/soil | | | | | | | | | | | | | | | | | | | | | | | | | | | | |  | |

Environmental Exposure in the LAST week

| Have you: |  |  |  |
| --- | --- | --- | --- |
| Walked in a paddock/field where farm animals graze? | Y | N | Not sure |
| Taken any day trips (beach, countryside, etc)?: | Y | N | Not sure |
| Had contact with wildlife species or their droppings?: | Y | N | Not sure |
| Had contact with soil, manure or sewage?: | Y | N | Not sure |

# Instructions for Healthy Adult Participant Questionnaire

These instructions will help you complete this questionnaire. Where possible, the question to which the instructions refer is in **bold**.

**Please write dates in dd/mm/yy format**

# Personal Details

The Unique Identifying Number (UIN) can be found on the invitation letter included with the questionnaire.

Please include your **occupation**, regardless of whether it fits into the risk groups. If it DOES fit into the risk groups, indicate which ones, and add more details if necessary.

A **food handler** is any person whose work involves preparing or serving unwrapped or unpackaged foods not subjected to further heating. It does not include supermarket checkout operators or those employed exclusively in the handling of packaged goods, delivery and the wholesale/retail sale of fruit or vegetables.

**Childcare setting** includes nurseries, day-schools, child minder, kindergartens, pre-school tutoring, and nannies.

A person who **has difficulty maintaining personal hygiene,** perhaps due to an unsatisfactory toilet, hand-washing or hand drying facilities at home, work or school.

**Work in contact with faeces** includes farm workers, people working with animals, sewage workers, specimen collection etc.

# Travel in the week prior to Completing Questionnaire

If more than two **countries/regions** were visited, they can be listed at the end of the form. Similarly for **towns/resorts.**

# Water exposure in the week prior to Completing Questionnaire

**Details** of water supplies include general locations or names of private water supplies

**Details** of water activities include locations etc.

# Animal Contact in the week prior to Completing Questionnaire

**Contact with animals** refers to ANY contact with animals – either at home, someone else’s home or any locations visited.

**Farm/petting zoo** includes open farms, studs, etc. as well as private farms.

**Handling** refers to leading around on a rope, corralling, grooming etc.

**Details for washing hands** include whether it was a bucket of standing water, the temperature, whether there was soap, drying facilities (paper vs. fabric towels) etc.

Please specify whether **consumption of food whilst there** occurred before, during, or after contact with the animals.

# Environmental Exposure in the week prior to Completing Questionnaire

**Walked in a paddock/field where farm animals graze** includes walking the dog, recreationally, through farm land.

**Day trips** refer to: beach/countryside/parks etc.

**Wildlife species** include: mice, deer, rabbits, foxes etc.

**Contact with manure** **or muck** may occur through activities such as gardening, DIY, etc. – includes attendance at agricultural shows etc.
